# Efficient Micropropagation of *Bistorta amplexicaulis* (D.Don) Greene: An Important Medicinal Plant of Western Himalaya

**DOI:** 10.1155/ianc/4457383

**Published:** 2025-01-29

**Authors:** Mudasir Fayaz, Seema Singh, Irshad Ahmad Bhat, Musadiq Hussain Bhat, Firdous Ahmad Khanday, Alamgir Ahmad Dar

**Affiliations:** ^1^Department of Botany, Plant Tissue Culture Research Laboratory, University of Kashmir, Srinagar 190006, Jammu and Kashmir, India; ^2^Division of Fruit Science, Sher‐e‐Kashmir University of Agricultural Sciences and Technology (SKUAST‐K), Srinagar 190025, Jammu and Kashmir, India; ^3^Department of Biotechnology, Signal Transduction Laboratory, University of Kashmir, Srinagar 190006, Jammu and Kashmir, India; ^4^Research Center for Residue and Quality Analysis, Sher‐e‐Kashmir University of Agricultural Sciences and Technology (SKUAST‐K), Srinagar 190025, Jammu and Kashmir, India

**Keywords:** amplexicine, mountain fleece, Polygonaceae, propagation

## Abstract

*Bistorta amplexicaulis* (D.Don) Greene from the family Polygonaceae is an important medicinal plant species. The growing therapeutic use of *B. amplexicaulis* has led to its population depletion thus requiring its conservation. Herein, an efficient, reproducible and reliable propagation protocol system was established for *B. amplexicaulis* using nodal segments as explant. Various culture media were tested for the assessment of growth and development of this plant species. On the shoot proliferation and rhizogenesis of regenerated *B. amplexicaulis* plantlets, the effects of several plant growth regulators (PGRs) were assessed. Direct organogenesis from nodal segments was achieved by culturing the nodal explants on Murashige and Skoog medium supplemented with 2.0 mg·L^−1^ 6-benzylaminopurine (BAP). Shoot multiplication was widely affected by the kind and concentration of PGRs, and the subculturing of in vitro regenerated shootlets on fresh medium. After incubation for 4 weeks at optimum BAP concentration, cultures were transferred to secondary medium with BAP (optimized concentration) supplemented with different auxins (indole acetic acid, indole butyric acid, and naphthalene acetic acid [NAA]). Murashige and Skoog medium enriched with 2.0 mg·L^−1^ BAP showed the highest shoot induction response (83% ± 3.61%) with mean shoot number (4.67 ± 1.45) and shoot length of 4.33 ± 1.45 cm. Growth medium fortified with 1.0 mg·L^−1^*α*- NAA exhibited maximum rhizogenesis with 4.33 ± 0.88 roots and average root length as 5.50 ± 0.76 cm from regenerated *B. amplexicaulis* shoots. Acclimatized plants of *B. amplexicaulis* showed 90% survival. The projected protocol may serve as a treasured tool for the rapid and large-scale multiplication of elite *B. amplexicaulis* and for germplasm conservation to ensure continuous supply of this plant amid the increasing demand.

## 1. Introduction

*Bistorta amplexicaulis* (D.Don) Greene is an erect rhizomatous herbaceous plant of 30–80 cm height. This plant prefers mountain slopes, moist places, and meadows. It is a medicinally important plant species from Polygonaceae native to Asia and is mostly found in Afghanistan, Nepal, China, India, and Pakistan [1, 2]. It is renowned for its medicinal properties, offering relief from a range of ailments such as flu, fever, joint pain, dysentery, cancer, heart issues, ulcers, and gastrointestinal disorders [3–5]. Traditionally, leaves are used as a natural remedy for dysentery and wounds, roots to alleviate coughs and dysentery, and it is believed that consuming the entire plant may induce abortion. Numerous pharmacological studies on this plant have revealed its diverse range of benefits, including antitumor, anticancer, antioxidant, hepatoprotective, and antimicrobial properties [5–11]. Its rhizome has showed notable cytotoxic and antitumor effects [9]. The increasing use of *B. amplexicaulis* for preventive and therapeutic purposes has led to a rise in demand. However, as this plant species only grows in the wild, there is significant pressure on natural populations, leading to its threatened status and the risk of extinction if this pressure continues. Currently, the plant species is classified as vulnerable [12]. The advancement of tissue culture techniques holds promise for the production of various phytochemicals sourced from different plant species [13]. In the present study, the primary aim was to establish a proficient in vitro micropropagation method for *B. amplexicaulis*, addressing the pressing need for its conservation and large-scale multiplication. This paper presents the inaugural in vitro propagation technique for this particular plant species.

## 2. Materials and Methods

### 2.1. Source of Plant Material, Explant Type, and Sterilization

Mother plants of *B. amplexicaulis* were collected from Aabnar Khag, Jammu and Kashmir, India (33°58′31.46″ N and 74°30′ 55.42″ E), at 2555 m (asl) in August 2020. The plant species was established in the Botanical Garden of Kashmir University and identified by Akhter H. Malik at the Center for Biodiversity and Taxonomy, Kashmir University Herbarium (KASH), where its herbarium specimen was deposited (Voucher No. 3135) for future reference. Mature 1–1.5 cm nodal segments were procured from mature healthy plants maintained in the University Botanical Garden. For standardization of the protocol, several explants which include leaf, node, shoot tip, and rhizome were thoroughly washed under tap water continuously for several minutes. This was followed by 1% (v/v) Labolene detergent (HiMedia Labs. Pvt. Ltd, Mumbai, India) treatment followed by treatment with few drops of Tween-20 surfactant (SRL, Pvt. Ltd, Mumbai, India). These were then washed under tap water and finally washed under laminar air flow hood (Biobase) with double-distilled water. In the laminar cabinet, explants were first pasteurized by 70% ethanol for 2–3 min followed by 2% Bavistin solution (BASF India Ltd.) treatment for 5 min. These explants were then treated with chemical sterilant mercuric chloride (0.01%) for 1–2 min. The remainder of the sterilant was then washed off of the explants using double-distilled water. Finally, aseptic explants were used for inoculation.

### 2.2. Culture Medium Composition and Conditions

#### 2.2.1. Callusing and Indirect Shooting

Murashige and Skoog [14] medium was supplemented with varied concentrations (0 to 4.0 mg·L^−1^) of different plant growth regulators (PGRs) 2,4-dichlorophenoxyacetic acid (2,4-D), 6-benzylaminopurine (BAP), thidiazuron (TDZ), and kinetin (KIN). Response percentage, response time, color, and texture of callus were recorded after 4 weeks. Calli were treated with various PGRs of different concentrations (0 to 6.0 mg·L^−1^). Results for shoot number, response percentage, average length of shoot, and mean response time were noted. All the chemicals used during the course of work were procured from HiMedia Laboratories Private Limited, Mumbai, Maharashtra, India, and were of Plant Tissue Culture Grade (PTC Grade).

#### 2.2.2. Shoot Initiation and Direct Shoot Regeneration

MS medium was fortified with a range (0.5–3.0 mg·L^−1^) concentrations of BAP, 2,4-D, KIN, ZEA and TDZ, sucrose (3%) procured from HiMedia Labs as carbon source, and agar (0.8%) as gelling agent. Media pH was adjusted between 5.5 and 5.7 by adding HCl or NaOH (0.1 N) each. Approximately, 10–15 mL medium was transferred into culture tubes of 30 mL capacity or culture flasks (150 mL), prior to autoclaving for 15–30 min, and culture room conditions were set as 22 ± 4°C temperature and 55% ± 2% relative humidity under 16/8 h photoperiod under fluorescent light (3000–4000 lux). After inoculation, culture tubes or flasks were sustained in the incubation room for 4 weeks. In order to promote and lengthen shoot growth, regenerated shoots were transferred to new medium. Shooting initiation, root regeneration, and other parameters were measured on the second day after inoculation. Subculturing was done on the medium that showed advanced shoot multiplication.

#### 2.2.3. Shoot Multiplication and Elongation

The shootlets were further cultured on MS medium with either optimal BAP concentration alone or in combination with BAP (2.0 mg·L^−1^) and auxins IAA, IBA, and NAA (0.25 to 1.50 mg·L^−1^) to assess the influence of secondary medium composition along with optimum 2.0 mg·L^−1^ BAP on elongation and multiplication of shoots. These cultures were further subcultured every 4 weeks on similar freshly prepared medium and repeated to four subculturing cycles.

#### 2.2.4. Rooting, Acclimatization, and Hardening

For rhizogenesis, well developed shoots with 2–4 nodes were collected from cultures and transferred to half or full MS medium with sucrose (3%) and agar (0.8%) complemented with auxins (IAA, IBA, and NAA). To get rid of stuck-on remnants of agar, plantlets with fully formed roots were gently washed under running water. The plantlets were placed in plastic containers with PGRs equal parts of soil, sand, and vermicompost. These were maintained in a well-equipped greenhouse under controlled conditions. Watering was done at regular intervals. To retain humidity, pots were covered with plastic. Hardened plants were moved to earthen pots with typical garden soil after 28 days, where they were kept in the shade for further growth.

### 2.3. Experiment Design and Statistical Analysis

Every single experiment was run using a randomized block design. For each treatment, each experiment had 20 repetitions and was run three times. For each aspect, observations on different parameters, such as shoot induction, shoot number, shoot length, and rhizogenesis, were recorded. ANOVA was used to evaluate all of the results, and Duncan's multiple range test with 0.05% probability was used to determine the significance of any differences.

## 3. Results

### 3.1. Callusing and Indirect Shoot Regeneration

**Figure 1:**
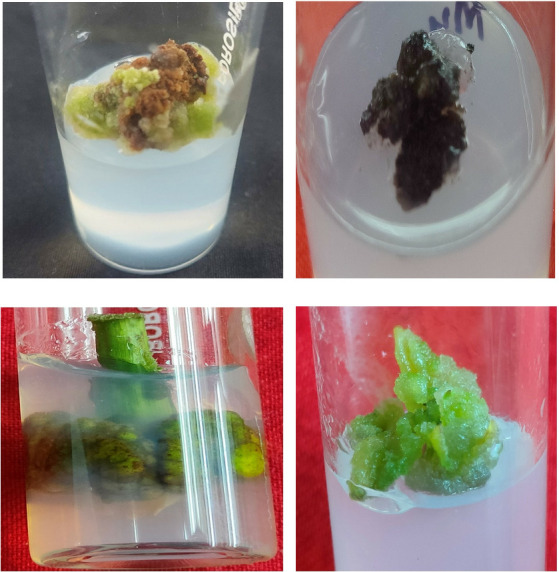
Callusing of *B. amplexicaulis* explants on (a) 2.0 mg·L^−1^ 2,4-D, (b) 2.0 mg·L^−1^ BAP, (c) 2.0 mg·L^−1^ TDZ, and (d) 2.5 mg·L^−1^ KIN.

Stem explant of *B. amplexicaulis* were inoculated on varied concentrations of different PGRs (Table 1). Out of the four PGRs used, 2,4-D showed the highest callusing (Figures 1(a), 1(b), 1(c), and 1(d)). Maximum callusing response (76.67% ± 3.33%) was detected in explants inoculated on MS medium with 2.0 mg·L^−1^ 2,4-D. Calli color observed was light brown and was hard (Table 1).

The increasing concentrations of 2,4-D positively influenced shoot response, shoot number, and shoot length up to a concentration of 5.0 mg/L, after which higher concentrations resulted in reduced shoot induction and growth. Optimal shoot morphogenesis for *B. amplexicaulis* was observed at 5.0 mg/L 2,4-D, with the highest number of shoots (12.00 ± 0.58) and longest shoot length (7.67 ± 0.33 cm) (Table 2).

### 3.2. Establishing Cultures and Regeneration of Direct Shoots

**Figure 2:**
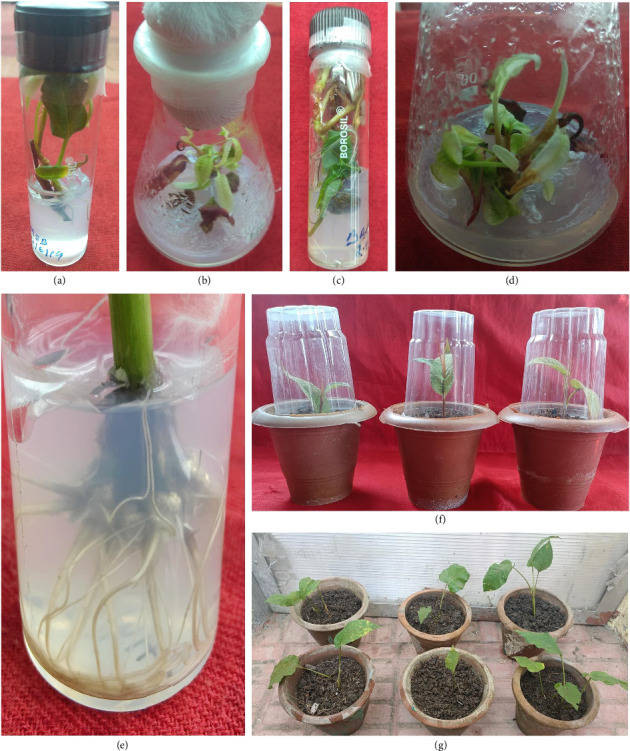
(a–c) Induction of shoots from the nodal explants on MS medium with 2.0 mg·L^−1^ 6-benzylaminopurine, (d) multiple shooting on MS medium + 2.0 mg·L^−1^ 6-benzylaminopurine and 1.0 mg·L^−1^ indole-3-acetic acid, (e) in vitro rhizogenesis from the shoots on full strength MS medium + 1.0 mg·L^−1^ naphthalene acetic acid, (f) hardening of plantlets of *B. amplexicaulis* in plastic cups, and (g) acclimatized plantlets.

MS medium augmented with 2.0 mg·L^−1^ BAP showed the highest regeneration (83% ± 3.61%) with a shoot number 4.67 ± 1.45 and length 4.33 ± 1.45 cm after 4 weeks of development (Table 3 and Figures 2(a), 2(b), and 2(c)). Shoot induction observed was high as compared to some previous reports for other members of family Polygonaceae (15, 16). Results for shoot induction were very low in cultures with MS medium fortified with other PGRs with low shoot length and number.

### 3.3. Impact of Various PGRs on the Development of New Shoots

Out of the different preparations used in the current study, combination of 2.0 mg·L^−1^ BAP and 1.0 mg·L^−1^ IAA enhanced the multiple shoot induction (85.22% ± 2.90%) with the highest shoot number (6.76 ± 1.14) and average shoot length (5.21 ± 0.91 cm) after 3 weeks incubation (Table 4 and Figures 2(d), 2(e), and 2(f)). Compared to other cytokinins, it is evident that BAP is the most effective one for rejuvenating shoots. The synergistic outcome of auxin and cytokinin blend for renewal of shoots showed the effectiveness order as IAA > NAA or IBA. All the auxins showed different rates of shoot induction at varied concentrations (0.25 to 1.0 mg·L^−1^). Though, auxin beyond 1.0 mg·L^−1^ concentration presented less shoot induction with average shoot number and length.

### 3.4. Role of Media Strength and Auxins in In Vitro Rooting

Among the tested MS media, full MS medium proved more beneficial. Results showed that the highest rooting (80%) with the maximum root number (12.67 ± 0.33) and root length (4.1 cm) was reported on full-strength medium augmented with 1.0 mg·L^−1^ NAA (Table 5 and Figures 2(g), 2(h), and 2(i)). In the present study, IBA or IAA was not as effective as NAA for rooting stimulation.

### 3.5. Acclimatization

After 30 days, 90% survival was observed in the rooted mature plantlets placed in the greenhouse potted in cups of plastic containing sand, soil, and vermicompost in 1: 1: 1 ratio. The micropropagated plants of *B. amplexicaulis* produced exhibited identical morphology and exempted from any apparent abnormality (Figures 2(f) and 2(g)).

## 4. Discussion

Establishing in vitro multiplication cultures for high-altitude plants, such as *B. amplexicaulis,* aids in both conservation and phytochemical production. Explants collected from June to August are ideal for this process, as their developmental stage during this period supports successful morphogenesis. This technique helps conserve medicinal plants and sustainably produce valuable compounds, benefiting biodiversity and pharmaceutical needs. There is no report on the indirect plant micropropagation system of *B. amplexicaulis.* This prompted the study mentioned herein using stem segments of mature plants as explant for initiation of callusing from which microshoots initiated. The main advantage of producing the indirect regeneration system of *B. amplexicaulis* is to produce disease-free plants. Similar studies have been carried out in many important medicinal plants such as *Jatropha curcas* [15], *Gymnema sylvestre* [16], and *Fragaria chiloensis* [17]. Calli were cultured on MS medium supplemented with various concentrations of PGRs for organogenesis. Given the distinct functions of these PGRs in cell division and other critical aspects of morphogenesis [18], stem segment–induced callus established on MS medium enriched with different PGRs produced shoots at varying levels within 15 days of culture. The number, length, and percentage of microshoot formation varied with the increased culture period. Among the four PGRs, 2,4-D was found to be the most effective, followed by BAP, KIN, and TDZ. Though, increased concentrations of the PGRs showed repressive effects on morphogenesis. Similar results have been observed in many plants such as *G. sylvestre* [16]. However, in shoot tip cultures of few plants such as *Capsicum chinense* [19] and callus cultures of *Solanum melongena*, callusing was accompanied by microshoot primordial induction with TDZ as most active PGR among the others [19, 20]. Nodal segments are considered the preferred type of explant and have been used in numerous studies of medicinal plants for shoot initiation [21–25]. Such explant type exhibit superior regeneration potential without any alterations in genetic stability [22]. It is found that the rate of shoot induction is usually high in the culture medium fortified with BAP when compared to other cytokinins, viz., 2,4-D, KIN, TDZ, and ZEA. Similar results have been reported in various micropropagation studies carried out on important medicinal plants which include *Bacopa monnieri, Dioscorea bulbifera, Eclipta alba*, *Ceropegia evansii*, *Morinda citrifolia*, and *Stevia rebaudiana* [26–31]. BAP is most prominent among the cytokinins due to its stable structure and its rapid assimilation by plant cells. The cytokinin–auxin ratio in the culture medium largely influences both shoot induction and differentiation. Such a synergistic effect of cytokinin and auxin combinations was also noticeable in the present study. A high cytokinin to lower auxin ratio exhibited enhanced shoot initiation and multiplication in *B. amplexicaulis.* Present studies revealed that BAP is clearly apparent to be the efficient cytokinin for regeneration of shoots in comparison to others. These verdicts are in promise to those reported by Lupi et al. [32] in *Helianthus annuus*. The synergistic effect of BAP in conjunction with auxins in varying concentrations has been confirmed in *in vitro* studies of several plant species of economically fruitful from the genus *Bistorta* and *Polygonum* [33]. In agreement with these records, the findings of this study also exhibited amplified shoot induction on MS medium added by cytokinin and auxin combinations. The effectiveness order of synergistic of cytokinin–auxin combination on regeneration of multiple shoots in *B amplexicaulis* was found as IAA > NAA or IBA. The positive effectiveness of IAA as compared to other auxins used has been noticed also in *C. evansii* [29] and *Nilgiranthus ciliatus* [34]. All the used auxins exhibited varying effects on multiple shoot regeneration rates at differentiations. But at higher concentrations (> 0.93 mg/L) of auxins, a decline in the shoot induction rate was observed. *C. evansii* have showed similar results where BAP in combination with IAA revealed the synergistic effect on regeneration frequency [29]. For rhizogenesis, among the different MS media strengths tested, full-strength medium showed enhanced results. *Philodendron bipinnatifidum* cultures also showed remarkable positive effects when rooting treatment was given in full-strength MS medium [35]. In the present study, NAA proved most beneficial and showed a remarkable enhanced effect on root stimulation as compared to IBA or IAA. Several plant species, for example, *Philodendron bipinnatum, Abutilon ranadei*, and *Nopalxochia ackermannii* exhibited similar results [35–37]. Our research introduces the initial micropropagation protocol for *B. amplexicaulis*, showcasing noteworthy results that could offer a viable solution for its mass production. Further investigation is needed in future to examine plant samples from mother plants and regenerated ones for their phytochemical quantification utilizing marker compounds to fix standards for *B. amplexicaulis*.

## 5. Conclusions

The findings of the current study revealed the synergistic effect of BAP and IAA combinations in the micropropagation system of *B. amplexicaulis* with enhanced shoot initiation and their proliferation. Optimized culture room conditions along with controlled supply of PGRs for maximum response led to the development of reliable, efficient, and economical micropropagation system for this important Himalayan plant species. This may possibly reduce both time and cost of large-scale plantlet production and may act as a beneficial tool for the regular availability of this important vulnerable seasonal plant species throughout the year and with proper genetic stability. Moreover, the developed protocol may also be employed for the enhanced production of some vital bioactive metabolites. Current findings also revealed that this micropropagation approach may pave a way to identify, mark, and standardize its bioactive components for novel drug discoveries for future studies.

## Figures and Tables

**Table 1:** Effect of various PGRs in varied combinations and concentrations on callus production from stem explant.

MS medium + PGR's (mg·L^−1^)		Response (% ± SE)	Number of days taken for callus production (mean ± SE)	Color and texture of callus
MS Basal		0.00 ± 0.00^a^	0.00 ± 0.00^a^	—

2,4-D	0.5	40.00 ± 5.78^b^	27.00 ± 1.15^d^	Brown and hard
	1.0	46.67 ± 6.67^bcd^	23.00 ± 1.15^bc^	Brown and hard
	1.5	51.67 ± 1.67^cd^	21.00 ± 0.58^b^	Light brown and hard
	2.0	76.67 ± 3.33^e^	24.00 ± 1.15^c^	Light brown and hard
	2.5	68.33 ± 1.67^e^	24.00 ± 0.58^c^	Brown and hard
	3.0	56.67 ± 3.33^d^	21.00 ± 0.58^b^	Brown and hard
	3.5	44.00 ± 0.58^bc^	25.33 ± 1.20^cd^	Brown and hard
	4.0	0.00 ± 0.00^a^	0.00 ± 0.00^a^	—

BAP	0.5	40.00 ± 5.78^b^	25.00 ± 2.89^b^	Dark brown and hard
	1.0	46.67 ± 3.53^bc^	24.33 ± 3.18^b^	Dark brown and hard
	1.5	63.33 ± 3.33^d^	25.67 ± 2.60^b^	Dark brown and hard
	2.0	73.33 ± 3.33^d^	23.00 ± 1.53^b^	Dark brown and hard
	2.5	70.00 ± 2.89^d^	23.67 ± 2.19^b^	Dark brown and hard
	3.0	51.67 ± 4.06^c^	23.67 ± 2.03^b^	Dark brown and hard
	3.5	0.00 ± 0.00^a^	0.00 ± 0.00^a^	—
	4.0	0.00 ± 0.00^a^	0.00 ± 0.00^a^	—

TDZ	0.5	40.00 ± 2.08^b^	29.00 ± 1.15^e^	Dark green and hard
	1.0	46.67 ± 3.33^c^	27.00 ± 1.15^cde^	Dark green and hard
	1.5	60.00 ± 1.73^d^	28.00 ± 0.58^de^	Dark green and hard
	2.0	70.00 ± 2.88^e^	24.00 ± 1.15^b^	Dark green and hard
	2.5	63.33 ± 3.33^d^	25.00 ± 1.15^bc^	Dark green and hard
	3.0	60.00 ± 2.52^d^	26.00 ± 1.15^bcd^	Dark green and hard
	3.5	0.00 ± 0.00^a^	0.00 ± 0.00^a^	—
	4.0	0.00 ± 0.00^a^	0.00 ± 0.00^a^	—

KIN	0.5	30.00 ± 2.89^b^	26.67 ± 1.45^e^	Light green and soft
	1.0	40.00 ± 2.52^c^	23.33 ± 0.88^d^	Light green and soft
	1.5	50.00 ± 1.73^d^	23.67 ± 1.45^d^	Green and hard
	2.0	58.33 ± 4.41^e^	20.33 ± 0.88^bc^	Green and hard
	2.5	66.67 ± 1.33^f^	18.33 ± 0.33^b^	Green and hard
	3.0	53.33 ± 3.84^de^	21.33 ± 0.88^cd^	Green and hard
	3.5	0.00 ± 0.00^a^	0.00 ± 0.00^a^	—
	4.0	0.00 ± 0.00^a^	0.00 ± 0.00^a^	—

*Note:* The significance of fluctuations in concentrations was calculated using DMRT, and the mean separation was analyzed by ANOVA using SPSS (*p* ≤ 0.05%). The small superscript letters (e.g., a, b, c, etc.) in the table represent statistical significance based on a post hoc test, commonly conducted after an Analysis of Variance (ANOVA). These letters are used to group means that are not significantly different from each other. Here's what they indicate: Same Letter: If two or more treatments share the same letter, it means their means are not significantly different at a specific confidence level (usually *p* < 0.05). For example, in the “Response (% ± SE)” column: MS Basal (0.00 ± 0.00a) and 4.0 (0.00 ± 0.00a) for 2,4-D are not significantly different from each other. Different Letters: If treatments have different letters, their means are significantly different. For example, for 2,4-D: 0.5 mg/L (40.00 ± 5.78b) is significantly different from 2.0 mg/L (76.67 ± 3.33e) in “Response (% ± SE)”. Combinations of Letters (e.g., bcd): This occurs when a treatment is not significantly different from multiple groups. For instance: 1.0 mg/L of 2,4-D (46.67 ± 6.67bcd) overlaps statistically with the b, c, and d groups. These letters provide a quick visual summary of which treatments are statistically similar or different based on the response or variable measured. This method helps interpret complex datasets efficiently.

**Table 2:** Effect of PGR (2,4-D) in varied concentrations on indirect shoot morphogenesis from stem-induced callus cultures of *B. amplexicaulis* cultivated for 4 weeks on MS medium.

Concentration (mg·L^−1^)	Response (% ± SE)	Shoot number/explant (Mean ± SE)	Shoot length (Mean ± SE)	No. of days (Mean ± SE)
0	0.00 ± 0.00^a^	0.00 ± 0.00^a^	0.00 ± 0.00^a^	0.00 ± 0.00^a^
0.5	21.67 ± 1.67^b^	2.00 ± 0.58^b^	0.23 ± 0.03^ab^	29.00 ± 1.73^de^
1.0	32.00 ± 1.00^c^	2.33 ± 0.67^bc^	0.60 ± 0.10^ab^	27.00 ± 1.15^bcde^
1.5	36.33 ± 2.03^cd^	2.67 ± 0.33^bcd^	0.77 ± 0.07^b^	28.00 ± 0.58^cde^
2.0	40.67 ± 0.67^de^	3.00 ± 0.58^bcd^	0.87 ± 0.03^bc^	25.00 ± 2.31^bcd^
2.5	42.33 ± 1.20^def^	3.33 ± 0.33^bcde^	0.93 ± 0.03^bc^	23.00 ± 1.73^b^
3.0	46.67 ± 1.67^ef^	3.67 ± 0.33^cde^	1.50 ± 0.29^c^	24.00 ± 2.31^bc^
3.5	48.00 ± 0.58^fg^	4.00 ± 0.58^de^	2.83 ± 0.17^d^	25.67 ± 1.76^bcd^
4.0	53.33 ± 3.33^gh^	6.00 ± 0.58^f^	3.33 ± 0.33^d^	26.00 ± 0.58^bcd^
4.5	60.00 ± 2.89^i^	8.33 ± 0.33^g^	4.67 ± 0.33^e^	27.00 ± 1.15^bcde^
5.0	75.00 ± 2.89^j^	12.00 ± 0.58^h^	7.67 ± 0.33^g^	23.00 ± 1.73^b^
5.5	58.33 ± 1.67^hi^	7.67 ± 0.33^g^	5.33 ± 0.33^f^	28.33 ± 0.67^cde^
6.0	55.00 ± 2.89^hi^	4.67 ± 0.33^ef^	4.50 ± 0.29^e^	31.33 ± 1.45^e^

*Note:* The significance of fluctuations in concentrations was calculated using DMRT, and the mean separation was analyzed by ANOVA using SPSS (*p* ≤ 0.05%). The small superscript letters (e.g., a, b, c, etc.) in the table represent statistical significance based on a post hoc test, commonly conducted after an Analysis of Variance (ANOVA). These letters are used to group means that are not significantly different from each other. Here's what they indicate: Same Letter: If two or more treatments share the same letter, it means their means are not significantly different at a specific confidence level (usually *p* < 0.05). For example, in the “Response (% ± SE)” column: MS Basal (0.00 ± 0.00a) and 4.0 (0.00 ± 0.00_a_) for 2,4-D are not significantly different from each other. Different Letters: If treatments have different letters, their means are significantly different. For example, for 2,4-D: 0.5 mg/L (40.00 ± 5.78_b_) is significantly different from 2.0 mg/L (76.67 ± 3.33_e_) in “Response (% ± SE)”. Combinations of Letters (e.g., bcd): This occurs when a treatment is not significantly different from multiple groups. For instance: 1.0 mg/L of 2,4-D (46.67 ± 6.67_bcd_) overlaps statistically with the b, c, and d groups. These letters provide a quick visual summary of which treatments are statistically similar or different based on the response or variable measured. This method helps interpret complex datasets efficiently.

**Table 3:** Effect of various PGRs in varied concentrations on shoot induction from nodal explants of *B. amplexicaulis.*

Concentration of cytokinin (mg·L^−1^)					Frequency of regeneration (% ± SE)	Shoot number (mean ± SE)	Shoot length (cm) (mean ± SE)
BAP	2,4-D	TDZ	KIN	ZEA			
0	0	0	0	0	0.00 ± 0.00^a^	0.00 ± 0.00^a^	0.00 ± 0.00^a^
0.5	—	—	—	—	46.67 ± 3.33^b^	3.67 ± 1.20^ab^	5.67 ± 1.76^b^
1.0	—	—	—	—	50.00 ± 5.77^b^	5.33 ± 1.45^b^	4.33 ± 0.88^ab^
1.5	—	—	—	—	65.00 ± 4.04^d^	3.67 ± 0.88^ab^	5.33 ± 2.03^b^
2.0	—	—	—	—	83.00 ± 3.61^d^	4.67 ± 1.45^b^	4.33 ± 1.45^ab^
2.5	—	—	—	—	80.00 ± 5.77^c^	5.33 ± 2.03^b^	5.33 ± 1.45^b^
3.0	—	—	—	—	53.33 ± 3.33^bc^	4.00 ± 1.53^ab^	3.33 ± 0.88^ab^
—	0.5	—	—	—	40.00 ± 5.78^b^	5.67 ± 1.76^b^	3.00 ± 1.15^ab^
—	1.0	—	—	—	56.67 ± 3.33^c^	4.00 ± 1.15^ab^	4.00 ± 1.15^ab^
—	1.5	—	—	—	70.00 ± 5.78^d^	4.67 ± 1.45^b^	4.16 ± 1.92^ab^
—	2.0	—	—	—	74.33 ± 0.88^d^	5.67 ± 0.88^b^	4.16 ± 1.59^ab^
—	2.5	—	—	—	72.33 ± 1.20^d^	5.33 ± 2.03^b^	4.67 ± 1.20^ab^
—	3.0	—	—	—	68.00 ± 0.58^d^	5.33 ± 1.45^b^	5.67 ± 1.76^b^
—	—	0.5	—	—	31.67 ± 4.41^b^	1.33 ± 0.33^ab^	0.97 ± 0.14^b^
—	—	1.0	—	—	46.67 ± 3.33^c^	1.33 ± 0.33^ab^	0.80 ± 0.10^b^
—	—	1.5	—	—	46.67 ± 3.33^c^	1.67 ± 0.33^b^	0.70 ± 0.15^b^
—	—	2.0	—	—	65.00 ± 2.87^d^	2.00 ± 0.58^b^	0.93 ± 0.12^b^
—	—	2.5	—	—	70.00 ± 5.77^d^	2.67 ± 0.67^b^	1.5 ± 0.29^c^
—	—	3.0	—	—	66.67 ± 4.41^d^	2.00 ± 0.58^b^	1.87 ± 0.08^c^
—	—	—	0.5	—	46.67 ± 3.33^b^	2.00 ± 0.58^b^	2.47 ± 0.80^bc^
—	—	—	1.0	—	50.00 ± 5.77^b^	1.67 ± 0.33^b^	2.03 ± 0.24^bc^
—	—	—	1.5	—	53.33 ± 4.41^b^	3.00 ± 0.58^b^	3.67 ± 0.58^c^
—	—	—	2.0	—	56.67 ± 3.33^bc^	2.67 ± 0.33^b^	3.23 ± 0.39^bc^
—	—	—	2.5	—	66.67 ± 3.33^cd^	2.33 ± 0.33^b^	1.77 ± 0.19^b^
—	—	—	3.0	—	70.00 ± 2.89^d^	2.67 ± 0.88^b^	2.20 ± 0.41^bc^
—	—	—	—	0.5	36.67 ± 1.67^c^	1.67 ± 0.33^b^	0.63 ± 0.22^b^
—	—	—	—	1.0	28.33 ± 1.67^b^	1.33 ± 0.33^b^	0.47 ± 0.12^b^
—	—	—	—	1.5	28.33 ± 4.41^b^	2.00 ± 0.58^b^	0.60 ± 0.23^b^
—	—	—	—	2.0	35.00 ± 2.89^bc^	1.67 ± 0.67^b^	0.53 ± 0.09^b^
—	—	—	—	2.5	0.00 ± 0.00^a^	0.00 ± 0.00^a^	0.00 ± 0.00^a^
—	—	—	—	3.0	0.00 ± 0.00^a^	0.00 ± 0.00^a^	0.00 ± 0.00^a^

*Note:* The significance of fluctuations in concentrations was calculated using DMRT, and the mean separation was analyzed by ANOVA using SPSS (*p* ≤ 0.05%). The small superscript letters (e.g., a, b, c, etc.) in the table represent statistical significance based on a post hoc test, commonly conducted after an Analysis of Variance (ANOVA). These letters are used to group means that are not significantly different from each other. Here's what they indicate: Same Letter: If two or more treatments share the same letter, it means their means are not significantly different at a specific confidence level (usually *p* < 0.05). For example, in the “Response (% ± SE)” column: MS Basal (0.00 ± 0.00_a_) and 4.0 (0.00 ± 0.00_a_) for 2,4-D are not significantly different from each other. Different Letters: If treatments have different letters, their means are significantly different. For example, for 2,4-D: 0.5 mg/L (40.00 ± 5.78b) is significantly different from 2.0 mg/L (76.67 ± 3.33e) in “Response (% ± SE)”. Combinations of Letters (e.g., bcd): This occurs when a treatment is not significantly different from multiple groups. For instance: 1.0 mg/L of 2,4-D (46.67 ± 6.67_bcd_) overlaps statistically with the b, c, and d groups. These letters provide a quick visual summary of which treatments are statistically similar or different based on the response or variable measured. This method helps interpret complex datasets efficiently.

**Table 4:** Effect of various PGRs in varied combinations and concentrations on shoot multiplication of *B. amplexicaulis* on MS medium.

BAP (mg·L^−1^)	IAA (mg·L^−1^)	IBA (mg·L^−1^)	NAA (mg·L^−1^)	Frequency of shoot multiplication (%±SE)	Shoot number (mean ± SE)	Shoot length (cm) (mean ± SE)
2.0	0.25	—	—	62.11 ± 2.21^b^	2.00 ± 0.58^a^	4.28 ± 0.40^a^
2.0	0.50	—	—	71.88 ± 2.40^c^	4.33 ± 0.88^ab^	4.23 ± 1.29^a^
2.0	0.75	—	—	70.19 ± 2.48^c^	5.67 ± 1.20^b^	4.67 ± 0.67^a^
2.0	1.0	—	—	85.22 ± 2.90^d^	6.76 ± 1.14^b^	5.21 ± 0.91^a^
2.0	1.25	—	—	61.39 ± 1.71^b^	4.28 ± 0.89^ab^	3.67 ± 0.44^a^
2.0	1.50	—	—	41.00 ± 2.65^a^	3.99 ± 0.58^ab^	3.21 ± 0.41^a^
2.0	—	0.25	—	46.67 ± 2.03^a^	2.00 ± 0.58^a^	1.87 ± 0.19^a^
2.0	—	0.50	—	67.12 ± 4.26^b^	4.21 ± 0.61^b^	3.31 ± 0.91^ab^
2.0	—	0.75	—	81.00 ± 3.00^c^	4.57 ± 0.72^b^	4.22 ± 0.76^b^
2.0	—	1.0	—	62.33 ± 1.86^b^	4.62 ± 0.69^b^	2.50 ± 0.50^ab^
2.0	—	1.25	—	61.67 ± 4.41^b^	3.22 ± 0.78^ab^	3.33 ± 0.67^ab^
2.0	—	1.50	—	58.33 ± 1.67^b^	2.67 ± 0.33^ab^	3.17 ± 0.17^ab^
2.0	—	—	0.25	40.00 ± 5.77^a^	3.00 ± 0.58^a^	1.30 ± 0.35^a^
2.0	—	—	0.50	46.67 ± 3.33^ab^	3.33 ± 0.67^a^	2.87 ± 0.41^b^
2.0	—	—	0.75	56.67 ± 4.41^bc^	2.67 ± 0.33^a^	1.83 ± 0.44^ab^
2.0	—	—	1.0	78.21 ± 3.75^d^	4.22 ± 1.13^a^	3.13 ± 0.19^b^
2.0	—	—	1.25	66.67 ± 3.33^cd^	3.33 ± 0.33^a^	2.67 ± 0.33^b^
2.0	—	—	1.50	63.33 ± 1.67^c^	2.33 ± 0.33^a^	3.00 ± 0.58^b^

*Note:* The significance of fluctuations in concentrations was calculated using DMRT, and the mean separation was analyzed by ANOVA using SPSS (*p* ≤ 0.05%). The small superscript letters (e.g., a, b, c, etc.) in the table represent statistical significance based on a post hoc test, commonly conducted after an Analysis of Variance (ANOVA). These letters are used to group means that are not significantly different from each other. Here's what they indicate: Same Letter: If two or more treatments share the same letter, it means their means are not significantly different at a specific confidence level (usually *p* < 0.05). For example, in the “Response (% ± SE)” column: MS Basal (0.00 ± 0.00_a_) and 4.0 (0.00 ± 0.00_a_) for 2,4-D are not significantly different from each other. Different Letters: If treatments have different letters, their means are significantly different. For example, for 2,4-D: 0.5 mg/L (40.00 ± 5.78_b_) is significantly different from 2.0 mg/L (76.67 ± 3.33_e_) in “Response (% ± SE)”. Combinations of Letters (e.g., bcd): This occurs when a treatment is not significantly different from multiple groups. For instance: 1.0 mg/L of 2,4-D (46.67 ± 6.67_bcd_) overlaps statistically with the b, c, and d groups. These letters provide a quick visual summary of which treatments are statistically similar or different based on the response or variable measured. This method helps interpret complex datasets efficiently.

**Table 5:** Effect of various PGRs in varied combinations and concentrations on in vitro root induction of *B. amplexicaulis* on full strength MS medium.

Auxin concentrations (mg·L^−1^)		Frequency of rooting (%±SE)	Root number (mean ± SE)	Root length (cm) (mean ± SE)
Control	0	0.00 ± 0.00^a^	0.00 ± 0.00^a^	0.00 ± 0.00^a^

IAA	0.25	0.00 ± 0.00^a^	0.00 ± 0.00^a^	0.00 ± 0.00^a^
	0.5	0.00 ± 0.00^a^	0.00 ± 0.00^a^	0.00 ± 0.00^a^
	0.75	43.33 ± 4.41^b^	4.33 ± 1.20^b^	2.50 ± 0.36^b^
	1.0	53.33 ± 3.33^c^	5.33 ± 0.88^b^	1.63 ± 0.68^b^
	1.25	0.00 ± 0.00^a^	0.00 ± 0.00^a^	0.00 ± 0.00^a^
	1.5	0.00 ± 0.00^a^	0.00 ± 0.00^a^	0.00 ± 0.00^a^

IBA	0.25	0.00 ± 0.00^a^	0.00 ± 0.00^a^	0.00 ± 0.00^a^
	0.5	0.00 ± 0.00^a^	0.00 ± 0.00^a^	0.00 ± 0.00^a^
	0.75	0.00 ± 0.00^a^	0.00 ± 0.00^a^	0.00 ± 0.00^a^
	1.0	40.00 ± 2.87^b^	3.67 ± 0.88^b^	1.43 ± 0.30^b^
	1.25	60.00 ± 2.52^c^	4.00 ± 0.58^b^	3.07 ± 0.64^c^
	1.5	53.33 ± 4.41^c^	3.00 ± 0.58^b^	2.63 ± 0.35^c^

NAA	0.25	20.00 ± 2.89^b^	2.00 ± 0.58^b^	1.80 ± 0.35^b^
	0.5	40.00 ± 1.73^c^	2.33 ± 0.33^b^	2.77 ± 0.39^bc^
	0.75	60.00 ± 2.52^e^	2.67 ± 0.88^b^	3.47 ± 0.43^c^
	1.0	80.00 ± 2.65^f^	12.67 ± 0.33^c^	5.50 ± 0.76^d^
	1.25	50.00 ± 2.08^d^	2.33 ± 0.33^b^	2.30 ± 0.35^bc^
	1.5	40.00 ± 2.31^c^	3.00 ± 0.58^b^	3.00 ± 0.51^bc^

*Note:* The significance of fluctuations in concentrations was calculated using DMRT, and the mean separation was analyzed by ANOVA using SPSS (*p* ≤ 0.05%). The small superscript letters (e.g., a, b, c, etc.) in the table represent statistical significance based on a post hoc test, commonly conducted after an Analysis of Variance (ANOVA). These letters are used to group means that are not significantly different from each other. Here's what they indicate: Same Letter: If two or more treatments share the same letter, it means their means are not significantly different at a specific confidence level (usually *p* < 0.05). For example, in the “Response (% ± SE)” column: MS Basal (0.00 ± 0.00_a_) and 4.0 (0.00 ± 0.00_a_) for 2,4-D are not significantly different from each other. Different Letters: If treatments have different letters, their means are significantly different. For example, for 2,4-D: 0.5 mg/L (40.00 ± 5.78_b_) is significantly different from 2.0 mg/L (76.67 ± 3.33_e_) in “Response (% ± SE)”. Combinations of Letters (e.g., bcd): This occurs when a treatment is not significantly different from multiple groups. For instance: 1.0 mg/L of 2,4-D (46.67 ± 6.67_bcd_) overlaps statistically with the b, c, and d groups. These letters provide a quick visual summary of which treatments are statistically similar or different based on the response or variable measured. This method helps interpret complex datasets efficiently.

## Data Availability

All data generated or analyzed during this study are included in this published article.
